# Atypical Bilateral Congenital Ectropion in a 3‐Day‐Old Female: A Case Report

**DOI:** 10.1155/crop/7578015

**Published:** 2026-11-10

**Authors:** Samidu Yahaya, Ida Ansing, Gilbert Bonsaana Batieka, Faisal Gunu Abdul-Samed

**Affiliations:** ^1^ Department of Surgery Ophthalmology Unit Tamale Teaching Hospital Tamale Ghana tamaleteachinghospital.org; ^2^ Department of Ophthalmology Ghana College of Physicians and Surgeons Accra Ghana; ^3^ Department of Ophthalmology School of Medicine University for Development Studies Tamale Ghana uds.edu.gh

**Keywords:** complete resolution, conservative treatment, ectropion, medicine, pediatrics

## Abstract

Bilateral congenital ectropion is an extremely rare neonatal eyelid anomaly. It is usually characterized by outward turning or eversion of the eyelid margins at birth, putting the affected neonates at a huge risk of exposure keratopathy. The global incidence remains unknown, with most literature limited to isolated case reports. In Ghana, published data are almost nonexistent on this condition. We report a 3‐day‐old female neonate who presented with bilateral congenital ectropion involving the upper eyelids. There were no associated congenital anomalies or systemic complications. Management consisted entirely of conservative treatment, including saline cleansing, ocular lubrication, reduction and pressure dressing, and parental education. Progressive improvement was noted within days, with complete resolution of the bilateral ectropion by Day 9 of conservative treatment. At review, 1 week following complete clinical resolution, the infant looked stable with normal lid and eyelash positions and a clear ocular surface bilaterally. She blinks to light in both eyes, and the mother reported no visual concerns, and there were no complications on subsequent follow‐up visits. The aim of this study is to highlight that bilateral congenital ectropion can be effectively managed using conservative measures, particularly in low‐resource settings where surgical intervention may not be readily accessible. Early recognition and timely conservative management are essential to achieving optimal outcomes.

## 1. Introduction

Ectropion is defined as the outward‐turning or eversion of the eyelid margin [1]. Congenital bilateral ectropion refers to the eversion of the eyelid margin present at birth and affecting both eyes. It is an extremely rare condition [2]. The true global incidence is unknown, with most literature reporting only isolated case reports and small case series [3]. Although bilateral congenital ectropion is rare globally, available data suggest a relatively higher incidence in Africans [4].

In Ghana, published data regarding the incidence or presentation of bilateral congenital ectropion are nonexistent at the time of this report, further stressing the rarity of this case, especially in Northern Ghana. The exact cause of bilateral congenital ectropion remains unclear, although it has been linked to some serious conditions, such as blepharophimosis syndrome, Down syndrome, and birth‐related trauma [4]. It may be linked to collodion infant syndrome (congenital ichthyosis) and has also been noted as an uncommon ocular feature of Down syndrome [5].

In this case report, we discuss the case of a 3‐day‐old female neonate who presented with congenital bilateral ectropion and achieved complete resolution in 9 days with conservative management in a low‐resource setting in the Northern Region of Ghana. This case highlights the effectiveness of conservative management and adds to the limited literature on this condition in Ghana.

## 2. Case Presentation

### 2.1. Case History/Examination

We report the case of a 3‐day‐old female neonate who was referred from a peripheral facility to the Tamale Teaching Hospital on account of congenital malformation of the eyelids (Figure 1). She was delivered as a late preterm neonate at 36+0 weeks via spontaneous vaginal delivery, with a birth weight of 2.8 kg. Her APGAR scores were 7 and 8, respectively. Her antenatal and perinatal periods were unremarkable, with no history suggestive of ichthyosis at birth, birth trauma, or prolonged labor.

**Figure 1:**
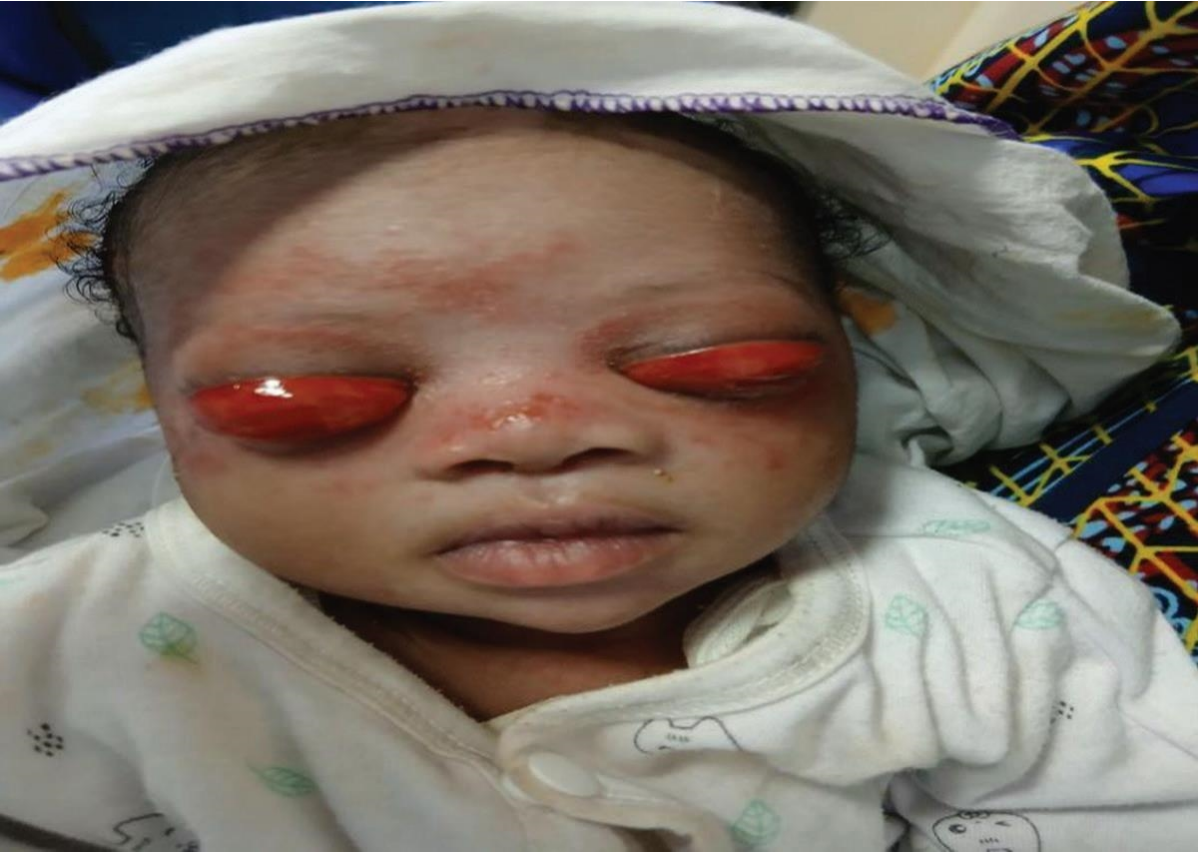
Bilateral congenital ectropion at presentation, demonstrating eversion of the eyelids with severe chemosis prior to initiation of conservative management.

On examination, she appeared stable and well‐hydrated. Vital signs were within normal limits for her age: oxygen saturation of 95% on room air, pulse rate of 121 bpm, temperature of 36.6°C, and respiration rate of 38 cycles/min. Anthropometric measurements showed a weight of 2.8 kg, a head circumference of 32 cm, and a length of 45 cm. Eye examination revealed bilateral upper eyelid eversion with severe chemosis of the palpebral conjunctiva. There was conjunctival prolapse. Slit‐lamp examination revealed clear corneas bilaterally, with no corneal epithelial defect, ulceration, or stromal thinning. There was no clinical evidence of exposure keratopathy at presentation. Complete eyelid closure was possible despite severe conjunctival chemosis and prolapse, which provided additional physical coverage of the cornea and may have reduced corneal exposure.

### 2.2. Differential Diagnosis, Investigations, and Treatment

She was diagnosed with bilateral congenital ectropion based on the history and eye examination. Conservative management was initiated immediately and consisted of the following protocol:

• Saline cleansing: The exposed conjunctiva was gently cleansed with gauze soaked in 0.9% isotonic sodium chloride solution every other day for 9 days.
• Ocular lubrication: Approximately 1% tetracycline ointment was applied every other day for 9 days to prevent dryness and corneal exposure.
• Manual reduction and pressure dressing: Following saline cleansing and ocular lubrication, manual repositioning of the ectropion was performed. Sterile gauze was then placed over the eyelid and secured with plaster to provide gentle pressure and maintain the eyelid in an anatomical position. This was performed on an alternate‐day basis.
• Parental education: Parents were educated on care, monitoring, and the nature of the condition to dispel superstitious beliefs that accompany congenital anomalies in Northern Ghana.

A pediatric review was conducted prior to the neonate′s presentation to the ophthalmology department to exclude associated congenital conditions, including ichthyosis and Down syndrome, and to rule out systemic involvement. Progressive improvement was noted with the above conservative management. By Day 7, the left upper eyelid ectropion had fully resolved (Figure 2), and by Day 9 (Figure 3), the right upper eyelid ectropion also resolved completely.

**Figure 2:**
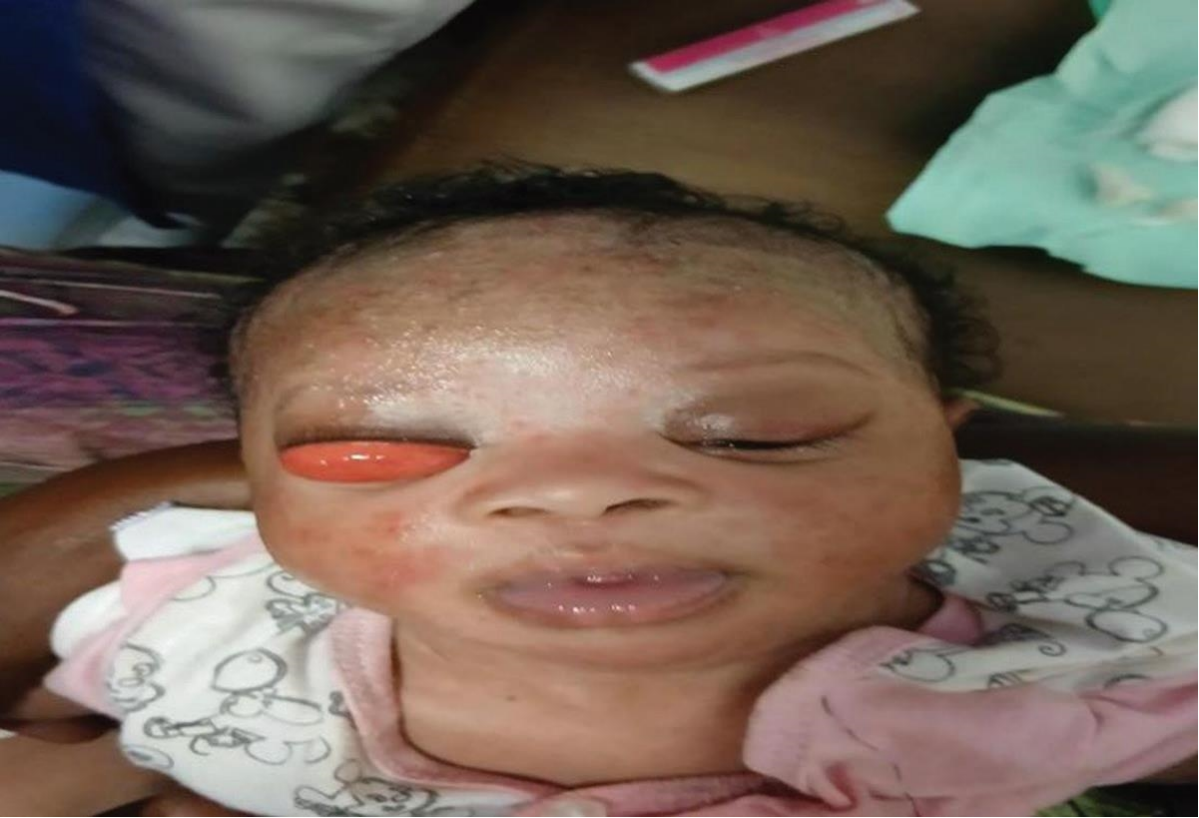
Day 7 of conservative management showing complete resolution of the left upper eyelid ectropion and almost normal lid position.

**Figure 3:**
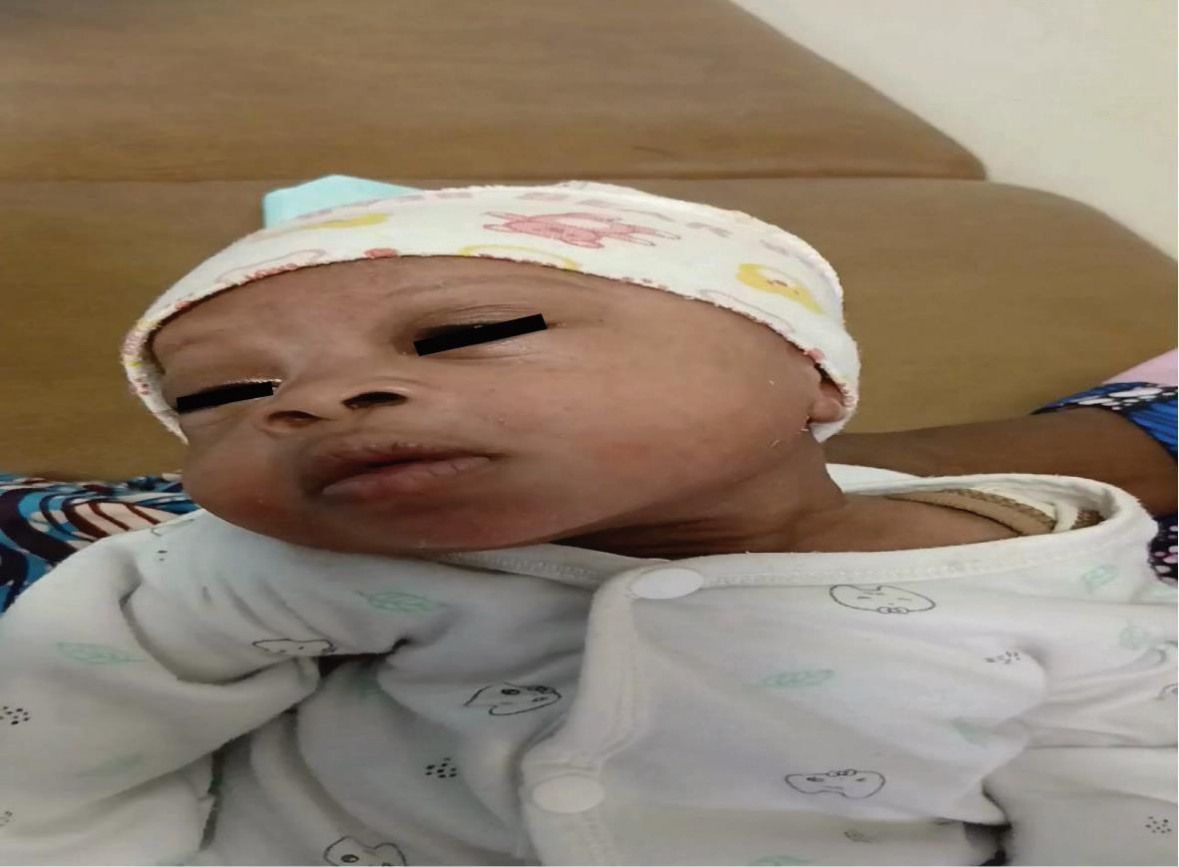
Day 9 of conservative management demonstrating complete resolution of both upper eyelid ectropions, with normal lid position.

## 3. Results and Conclusion

Bilateral congenital ectropion is an extremely rare neonatal eyelid anomaly. It is usually characterized by outward turning or eversion of the eyelid margins at birth, putting affected neonates at significant risk of exposure keratopathy. The global incidence remains unknown, with most literature limited to isolated case reports. In Ghana, published data on this condition are almost nonexistent. We report a 3‐day‐old female neonate who presented with bilateral congenital ectropion involving the upper eyelids. There were no associated congenital anomalies or systemic complications. Management consisted entirely of conservative treatment, including saline cleansing, ocular lubrication, manual reduction, and pressure dressing, along with parental education. Progressive improvement was noted within days, with complete resolution of the bilateral ectropion by Day 9 of conservative treatment. At review 1 week following complete clinical resolution, the infant appeared stable, with normal lid and eyelash position and a clear ocular surface bilaterally. Formal visual acuity assessment was not possible due to the patient′s neonatal age; the infant demonstrated a blink response to light, which was regarded as a gross behavioral response and not a measure of visual acuity. No clinical evidence of significant corneal opacity or other anterior segment abnormality was identified. No recurrence or ocular complications were observed at follow‐up at 1 and 6 months. Conservative management was successful in this case of bilateral congenital ectropion; hence, surgical intervention was not required. The rapid and complete resolution observed in this neonate supports conservative management as an initial approach, particularly in low‐resource settings where surgical intervention may not be readily accessible. Early recognition and timely conservative management may contribute to optimal outcomes. This case adds to the limited literature on this rare condition in Ghana.

## 4. Discussion

Congenital ectropion is an uncommon condition. It often affects the upper eyelid and manifests at birth. It can be characterized by total turning out of the eyelids margin and is generally accompanied by chemosis, conjunctival prolapse, and edema [6]. It is an extremely rare and benign condition of the eyelids [7]. Although the exact etiology still remains unclear, several theories have been hypothesized, such as orbicularis oculi hypotonia, vertical shortening of the anterior lamella, elongation of the posterior lamella, and failure of the orbital septum to fuse with the levator aponeurosis [8].

Globally, conservative management is still the initial and standard protocol, especially for cases that present early. For example, a case report highlighted how taping a neonate′s eyelid led to complete resolution [9]. Recent case reports described the use of gauze padding, hypertonic saline, antibiotics, and lubrication treatment, with complete resolution of the eyelids ectropion occurring in days to weeks [7, 10]. Another case of a normal newborn with bilateral upper eyelid eversion was successfully managed conservatively within 14 days by the application of topical antibiotic, steroid, artificial tears, and padding with normal‐saline‐soaked gauze [11].

The case presented in this report is similar to the above cases, which showed how congenital ectropion can completely resolve in a matter of weeks with only conservative measures. Although bilateral congenital ectropion is rare globally, a number of published case reports have described the condition in infants of African descent [7, 8]. For example, Ibraheem [8] reported a bilateral congenital ectropion in a 6‐day‐old Nigerian neonate who recovered fully within 5 days of conservative management, whereas Panshak et al. [12] reported about a 3‐day‐old neonate in Nigeria with unilateral congenital ectropion, which resolved completely within 3 days following conservative management with 3% hypertonic saline, antibiotic drops/ointment, and gauze padding. However, given the inherent limitations of case reports and the potential for publication bias, these observations cannot establish a racial predisposition. Further population‐based studies are needed to determine whether any true racial or ethnic variation in incidence exists.

Even though there are relatively multiple case reports from other parts of Africa, congenital ectropion‐specific data from Ghana are strikingly nonexistent. A review of congenital ocular anomalies in Ghana at the Korle Bu Teaching Hospital from 2004 to 2009 did not list congenital ectropion among the major anomalies. Instead, cataract, retinoblastoma, and glaucoma were more common [13]. This reflects the rarity or underreporting of congenital ectropion in Ghana. It also highlights a gap in national ophthalmologic literature and perhaps a need for pediatricians and ophthalmologists in the country to be more alert.

With the potential risk of exposure keratopathy and corneal ulceration, early detection and timely management are necessary [8]. Surgical options, such as tarsorrhaphy, are only resorted to when conservative management fails [12].

## 5. Conclusions

This case highlights that bilateral congenital ectropion can be effectively managed using conservative measures alone, particularly in low‐resource settings where surgical intervention may not be readily accessible. Early recognition and timely conservative management are essential to achieving optimal outcomes. In addition, given how rare bilateral congenital ectropion is and the scarcity of documented cases in Ghana, this case adds valuable data to the Ghanaian ophthalmic literature and underscores the need for increased awareness among neonatal and eye‐care providers to ensure optimal outcomes.

## 6. Limitation

This case report has several limitations. First, the single‐case design limits generalizability, and causality between the intervention and outcome cannot be established. Second, treatment efficacy cannot be definitively determined from a single case, as spontaneous resolution may occur. Third, no advanced technology was available to confirm associated anomalies beyond clinical examination. Fourth, objective assessment of visual function was not possible due to the patient′s neonatal age. Fifth, the duration of follow‐up, while extending to 6 months, does not allow assessment of long‐term visual outcomes or late complications. Despite these limitations, this case provides valuable insights into the clinical course and conservative management of this rare condition.

## Author Contributions

Samidu Yahaya: conceptualization, data curation, project administration, writing—original draft, writing—review and editing. Ida Ansing: writing—review and editing. Gilbert Bonsaana Batieka: writing—review and editing. Faisal Gunu Abdul‐Samed: writing—review and editing.

## Funding

No funding was received for this manuscript.

## Consent

The patient parents provided written informed consent for the publication of this case report.

## Conflicts of Interest

The authors declare no conflicts of interest.

## Data Availability

The data that support the findings of this study are available from the corresponding author upon reasonable request.
